# The harvest plot: A method for synthesising evidence about the differential effects of interventions

**DOI:** 10.1186/1471-2288-8-8

**Published:** 2008-02-25

**Authors:** David Ogilvie, Debra Fayter, Mark Petticrew, Amanda Sowden, Sian Thomas, Margaret Whitehead, Gill Worthy

**Affiliations:** 1 Medical Research Council Social and Public Health Sciences Unit, Glasgow, UK; 2 Centre for Reviews and Dissemination, University of York, York, UK; 3 Department of Public Health, University of Liverpool, Liverpool, UK; 4 Medical Research Council Epidemiology Unit, Cambridge, UK; 5 London School of Hygiene and Tropical Medicine, London, UK

## Abstract

**Background:**

One attraction of meta-analysis is the forest plot, a compact overview of the essential data included in a systematic review and the overall 'result'. However, meta-analysis is not always suitable for synthesising evidence about the effects of interventions which may influence the wider determinants of health. As part of a systematic review of the effects of population-level tobacco control interventions on social inequalities in smoking, we designed a novel approach to synthesis intended to bring aspects of the graphical directness of a forest plot to bear on the problem of synthesising evidence from a complex and diverse group of studies.

**Methods:**

We coded the included studies (n = 85) on two methodological dimensions (suitability of study design and quality of execution) and extracted data on effects stratified by up to six different dimensions of inequality (income, occupation, education, gender, race or ethnicity, and age), distinguishing between 'hard' (behavioural) and 'intermediate' (process or attitudinal) outcomes. Adopting a hypothesis-testing approach, we then assessed which of three competing hypotheses (positive social gradient, negative social gradient, or no gradient) was best supported by each study for each dimension of inequality.

**Results:**

We plotted the results on a matrix ('harvest plot') for each category of intervention, weighting studies by the methodological criteria and distributing them between the competing hypotheses. These matrices formed part of the analytical process and helped to encapsulate the output, for example by drawing attention to the finding that increasing the price of tobacco products may be more effective in discouraging smoking among people with lower incomes and in lower occupational groups.

**Conclusion:**

The harvest plot is a novel and useful method for synthesising evidence about the differential effects of population-level interventions. It contributes to the challenge of making best use of all available evidence by incorporating all relevant data. The visual display assists both the process of synthesis and the assimilation of the findings. The method is suitable for adaptation to a variety of questions in evidence synthesis and may be particularly useful for systematic reviews addressing the broader type of research question which may be most relevant to policymakers.

## Background

### Beyond the forest plot

In systematic reviews of the effects of interventions, the objective of synthesising evidence from multiple studies is often expressed in terms of seeking an overall conclusion about effectiveness. Guidance such as that produced by the Cochrane Collaboration or the Centre for Reviews and Dissemination (CRD) distinguishes between 'quantitative' methods of synthesis (particularly meta-analysis) and 'descriptive', 'non-quantitative' or 'narrative' methods of synthesis. For example, the Cochrane handbook describes the use of narrative synthesis 'where meta-analysis is either not feasible or not sensible', [1] and CRD guidelines refer to the possibility that 'a non-quantitative synthesis may informally explore how the differences in study characteristics affect their results' if meta-analysis is deemed not feasible [2].

One attraction of meta-analysis is that the results can be summarised using a graphical plot such as a forest plot, in which each study is represented by a square indicating the point estimate of the effect size and a horizontal line indicating the confidence interval around that estimate. The pooled estimate of the effect size and its confidence interval are represented by a diamond at the bottom of the figure. Forest plots thereby provide a compact, visually striking overview of the essential data from each individual study and the overall 'result' [3].

However, the statistical validity of meta-analysis depends on a degree of homogeneity between studies, not least in terms of their outcome metrics, [1,2] which may be unrealistic outside the world of clinical trials. For example, Slavin – originator of the concept of 'best evidence synthesis' – questions whether studies should be excluded solely because an effect size suitable for meta-analysis cannot be calculated from their results, and challenges the assumption that meta-analysis is necessarily the most meaningful way of synthesising data on effectiveness in the first place [4]. The guidelines of the Cochrane Health Promotion and Public Health Field warn that even if data are statistically amenable to meta-analysis, a systematic reviewer 'needs to make the case for meta-analysis before proceeding' [5].

A recent project aiming to produce guidance on alternative, 'narrative' methods of synthesis found that such methods do not rest on an authoritative body of knowledge [6]. Techniques used range from those typically associated with qualitative research, such as thematic analysis, [7] through a variety of tabular approaches, to the quantitative analysis and graphical plots of quantities such as odds ratios. By definition, however, narrative synthesis depends substantially on using text to 'tell the story' [6]. If the number of included studies is large, this can result in a lengthy and somewhat indigestible results section which may compare unfavourably with the brevity and immediacy of a forest plot.

### Seeking evidence about differential effects

The complexity of synthesis is increased if a systematic review examines multiple related research questions. The formation of the Cochrane Collaboration Health Equity Field and the Campbell Collaboration Equity Methods Group reflects a growing interest in synthesising evidence about how the effects of interventions vary between demographic and socio-economic groups [8]. Despite the political priority given to reducing health inequalities in recent years, few systematic reviews have yet examined either the effectiveness of interventions intended to reduce health inequalities or the distributional effects of interventions applied to whole populations. Understanding whether and how interventions work in different groups is important to ensure that apparently beneficial aggregate population effects do not conceal widening disparities in health between more and less advantaged groups [9].

There are, however, numerous dimensions of inequality, such as those enumerated in the PROGRESS criteria (place of residence, race or ethnic origin, occupation, gender, religion, education, socioeconomic status and social capital) [10]. Synthesising evidence about multiple potential social gradients in the effects of interventions therefore poses a methodological challenge for those conducting systematic reviews. We present a method which we devised in the course of a systematic review of the effects of population-level tobacco control interventions on social inequalities in smoking. We aimed to combine aspects of the graphical directness of a forest plot with a sufficient, but not exhaustive, narrative account of what could be learned from a highly diverse group of studies. Our method is not specific to the topic of the review and could readily be applied to, or adapted for, other research questions.

## Methods

### Input data

The general methods for the systematic review have been reported elsewhere (Thomas S, Fayter D, Misso K, Ogilvie D, Petticrew M, Sowden A, Whitehead M, Worthy G. Population tobacco control interventions and their effects on social inequalities in smoking: systematic review, submitted). Briefly, we searched widely for studies which had assessed the effects of any type of population-level tobacco control intervention and had reported effects stratified by at least one demographic or socio-economic characteristic. We included all studies meeting these criteria irrespective of study design, methodological quality or outcomes measured. We coded studies on two methodological dimensions: a three-point scale of suitability of study design, adapted from the criteria used for the Community Guide of the US Task Force on Community Preventive Services, [11] and a six-item checklist of quality of execution, adapted from the criteria developed for the Effective Public Health Practice Project in Hamilton, Ontario and designed to be applicable across the entire range of included study designs [12] (Additional file 1).

The characteristics of the included studies have also been reported elsewhere (Thomas S, Fayter D, Misso K, Ogilvie D, Petticrew M, Sowden A, Whitehead M, Worthy G. Population tobacco control interventions and their effects on social inequalities in smoking: systematic review, submitted). The 85 studies ranged from randomised controlled trials of measures to prevent tobacco from being sold to minors (those under the legal minimum purchase age) to cross-sectional econometric analyses of the price elasticity of demand for cigarettes and included a variety of other experimental and observational, controlled and uncontrolled study designs. The effects of interventions in this field have been assessed using a wide range of outcomes and outcome measures (often several within the same study) ranging from self-reported changes in awareness of no-smoking policies to directly-observed changes in smoking behaviour (Table 1). Across the included studies as a whole, effects have been stratified by six different dimensions of inequality – by income, occupation, education, gender, race or ethnicity, and also by age – but rarely by more than two or three of these dimensions within a single study.

**Table 1:** Examples of diverse outcome measures

**Outcome**	**Outcome measures**
Change in awareness	Awareness that an institution had a no-smoking policy Awareness that health warnings on cigarette packs had been changed
Change in attitude	Answer to the question 'Do you think young people who smoke have more friends?'
Change in perceived availability	Reported ease of obtaining cigarettes from shops
Change in self-reported smoking status	Prevalence of 'regular' smoking (at least one cigarette per day) Incidence of quitting

### Defining the hypotheses to be tested

We took a hypothesis-testing approach. For each study and each dimension of inequality, we specified a null hypothesis (that there was no social gradient in the effectiveness of the intervention) and two alternative hypotheses (one that there was a positive social gradient in effectiveness, and one that there was a negative social gradient in effectiveness). We defined a positive gradient in effectiveness as a situation in which the intervention was more effective in more advantaged groups (defined for this purpose as the more affluent, those with a higher level of education, those in more skilled occupational groups, males, older people, or those in the majority or most advantaged racial or ethnic group in the context of a particular study), whereas a negative gradient was defined as a situation in which the intervention was more effective in more disadvantaged groups. Since we were examining the evidence from an equity perspective, we were particularly keen to identify interventions with a negative gradient in effectiveness in order to inform policies to reduce inequalities in health.

### Allocating each study to the best supported hypothesis

For some studies – for example, those with a single outcome measure and an unambiguous finding that the intervention was more effective in certain groups than others – determining which of the competing hypotheses was best supported by that study was straightforward. However, some studies presented conflicting outcome data. In such cases, the pair of reviewers appraising each study had to reach an agreed overall judgment about how the results should be interpreted from the equity perspective, for example by giving greater weight to certain outcome measures.

For example, an econometric study by Chaloupka and Wechsler found a negative price elasticity of demand for cigarettes among both men and women [13]. However, the direction of the social gradient in price elasticity depended on how demand for cigarettes was defined. Women's participation in smoking (i.e. whether they had smoked in the last 30 days) was more sensitive to price than men's, whereas men's cigarette consumption (i.e. the quantity of cigarettes smoked) was more sensitive to price than women's. We categorised this study overall as best supporting the null hypothesis of no gradient in effectiveness by gender. Another econometric study by Lewit and colleagues found that participation in smoking at age 14 was more sensitive to price in boys than in girls, whereas the price elasticity of the intention to smoke (i.e. the perceived likelihood of taking up smoking in the next year) was similar in boys and girls [14]. We categorised this study as overall supporting the hypothesis of a positive gradient in effectiveness by gender, i.e. that an increase in price was more effective in males, and described the conflicting data in the text of the results section in our full report.

### Plotting the distribution of the evidence

**Figure 1:**
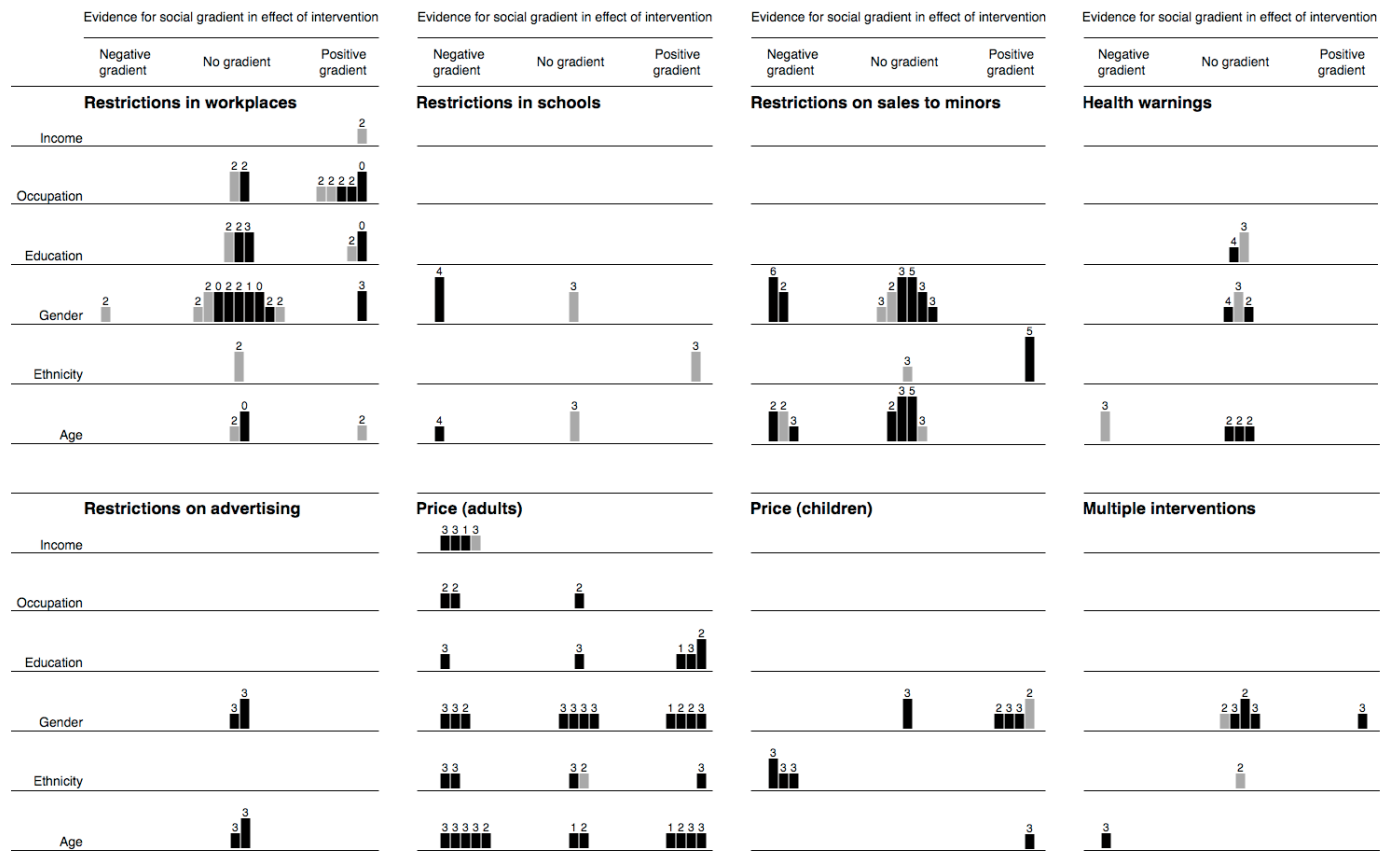
**Evidence for social gradients in effects of all categories of intervention**. A 'supermatrix' covering all categories of intervention consisting of six rows (one for each dimension of inequality) and three columns (one for each of the three competing hypotheses about the differential effects of each category of intervention). Each study is represented by a mark in each row for which that study had reported relevant results. Studies with 'hard' behavioural outcome measures are indicated with full-tone (black) bars, and studies with intermediate outcome measures with half-tone (grey) bars. The suitability of study design is indicated by the height of the bar. Each bar is annotated with the number of other methodological criteria (maximum six) met by that study. See *Methods: Plotting the distribution of the evidence *for further explanation.

For each category of intervention (such as restrictions on sales to minors) we then populated a matrix to show the distribution of the evidence, drawing on examples of the use of matrices to analyse and synthesise qualitative data [15]. Each matrix consisted of six rows (one for each dimension of inequality) and three columns (one for each of the three competing hypotheses). These matrices are reproduced as a single combined 'supermatrix' covering all categories of intervention (Figure 1). We represented each study with a mark in each row (dimension of inequality) for which that study had reported relevant results. To emulate the visual representation of study weighting in a forest plot, we weighted and annotated the marks for each study to indicate three characteristics:

▪ Studies with 'hard' behavioural outcome measures (changes in smoking behaviour) were indicated with full-tone (black) bars, and studies with intermediate outcome measures (such as changes in attitude) with half-tone (grey) bars

▪ The suitability of study design was indicated by the height of the bar

▪ Each bar was annotated with the number of other methodological criteria met by that study.

### Focused narrative synthesis

We then applied these matrices to the problem of synthesis in both formative and summative ways. On the one hand, we used the plots to identify areas of the evidence base on which to concentrate our narrative synthesis – for example, areas with the most compelling evidence for a positive or negative social gradient in effectiveness, or 'deviant' cases (isolated studies with apparently atypical or discordant results). On the other hand, we also used the plots to accompany the narrative synthesis in summarising our results – for example, to draw attention to the white space, which indicated the types of intervention and dimensions of inequality which had been least thoroughly researched, or to draw attention to the higher quality of evidence about, for example, the effects of restrictions on sales to minors compared with those of restrictions on smoking in schools.

## Results

**Figure 2:**
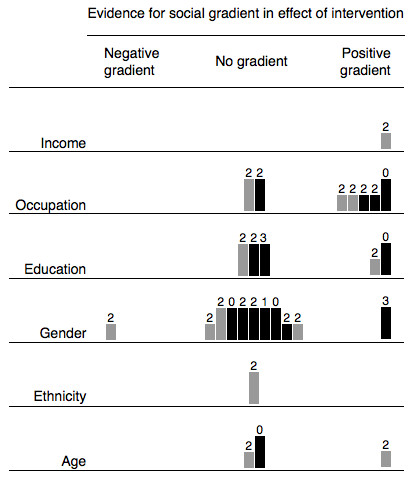
**Evidence for social gradients in effects of restrictions on smoking in workplaces and public places**. Each study is represented by a mark in each row for which that study had reported relevant results. Studies with 'hard' behavioural outcome measures are indicated with full-tone (black) bars, and studies with intermediate outcome measures with half-tone (grey) bars. The suitability of study design is indicated by the height of the bar. Each bar is annotated with the number of other methodological criteria (maximum six) met by that study. See *Methods: Plotting the distribution of the evidence *for further explanation.

The resulting matrices highlighted certain areas of the evidence base which appeared to be particularly relevant to our research question concerning differential effects, and helped to focus our narrative synthesis and discussion on the relevant topics. These are exemplified by the finding that increasing the price of tobacco products may be more effective in discouraging smoking among people with lower incomes and in lower occupational groups. We considered it equally important to identify interventions with the potential to increase inequalities as to identify those with the greatest potential to reduce them, and in this regard we found a somewhat reassuring absence of clear evidence for an adverse social gradient in the effects of many categories of intervention. Again, however, the matrices helped us to identify areas of possible concern. For example, the matrix for restrictions on smoking in workplaces and public places (Figure 2) suggests stronger evidence for a gradient in effectiveness by occupational group than by any other demographic or socio-economic characteristic. However, the distribution of the tones, heights and annotations of the bars (studies) populating this row of the matrix suggests that the evidence for such a gradient was mostly contributed by comparatively weak study designs, some of which found a gradient only in 'intermediate' rather than 'hard' outcome measures. By focusing on this group of studies, considering the context of the interventions in question, and drawing on related qualitative studies, we were able to synthesise our findings as: '... if anything, restrictions on smoking in workplaces [only] may be more effective for staff in higher occupational grades' (Thomas S, Fayter D, Misso K, Ogilvie D, Petticrew M, Sowden A, Whitehead M, Worthy G. Population tobacco control interventions and their effects on social inequalities in smoking: systematic review, submitted).

## Discussion

We have presented a novel method for synthesising evidence about the differential effects of heterogeneous and complex interventions. Unlike a forest plot, which highlights the 'bottom line' from the synthesis of a number of similar studies of similar interventions in similar participants, we do not see our matrices as providing a definitive statement of the 'results' of a systematic review; rather, they form part of the analytical process as much as they help to summarise the output. Nonetheless, early feedback from peer reviewers and conference delegates suggests that this method of displaying summary data does aid the assimilation of a complex set of findings. We propose the name 'harvest plot' for matrices of the kind we have demonstrated, reflecting the process of gathering and winnowing the best available evidence from all corners of the field.

### Advantages of the harvest plot

The first advantage of our method is that it is agnostic to the outcomes and metrics used in the primary studies. Slavin's critique of meta-analysis [4] is addressed by this method because no data need be discarded: all are relevant because all can be judged in terms of whether they tend to support a particular hypothesis or not. The method therefore helps to maximise, rather than constrain, the potential learning which can be derived from the studies included in a systematic review.

The second advantage is that the method can be tailored to those characteristics of studies which are most relevant within a particular body of evidence. In this review, we chose to emphasise the suitability of study design over the quality of execution of the studies because, having examined all the available evidence, we judged study design to be the more important metric on which to grade the weight to be attached to the findings of each study. As a consequence, the matrices make it particularly clear that large parts of the available evidence base depend wholly or partly on weak study designs, as well as on 'intermediate' outcome measures represented by the grey bars. Nonetheless, users who wish to know the number of methodological criteria met by particular studies can still find these data in the matrices. We also chose not to emphasise sample size (typically used as the primary weighting factor in a meta-analysis of commensurable studies) because we considered this characteristic to be incommensurable across all study designs included in this particular systematic review, which ranged from randomised controlled trials (with a typical sample size of the order of 10^2 ^to 10^3^) to econometric analyses of large population datasets (with a typical sample size of the order of 10^4 ^to 10^5^). Nonetheless, users who wish to know the sample sizes for particular studies can still find these data in the tables in the full report. It would be easy to adapt the principle of the harvest plot to reflect the nature of the available evidence for a different systematic review – for example by using the tone of the bars to distinguish randomised from non-randomised studies, or (if all included studies were of a similar study design) by using the height of the bars to represent sample size. However, researchers should bear in mind that choosing to emphasise different study characteristics may influence the interpretation of where the balance of best available evidence lies.

The third advantage is that, like any graphical method, the harvest plot can not only 'make the statistics a little more palatable' [16] but can also help us to 'discover what we were not expecting' [17]. In the process of synthesising the evidence, we found it easier to compare evidence between types of intervention, dimensions of inequality and competing hypotheses and to identify patterns of interest by examining the matrices than by studying lengthy tables filled with large quantities of text; users of reviews may also find that a visual display helps them to assimilate and digest the findings from a complex review. This is not, however, to deny the importance of extracting and tabulating all relevant data from the primary studies: the tables remain an essential component of the process and are needed both to validate and to interpret the patterns revealed by the harvest plot.

### Limitations of the harvest plot

One limitation is that a method which admits 'all comers' in terms of outcome metrics and, in this case, study designs is clearly more appropriate for some types of systematic review than others. It is likely to be particularly useful for systematic reviews conducted from a 'lumping' rather than a 'splitting' perspective – i.e. those addressing the broader questions which may be more relevant to policymakers [18]. In other situations, however, including such a wide range of data in the same matrix may run the risk of concealing, or even distorting, the most important and valid inferences which could be derived from a subset of the most robust studies.

Another limitation is the risk that more may be read into the matrices than is justified by the data, particularly if they are displayed on their own without the accompanying narrative synthesis and an account of the methodological limitations of the primary studies. For example, it is now common for speakers to be asked to provide their slides for posting on conference websites, but Tufte and others have highlighted the hazards of relying on this type of standalone 'slideument' (a slide show masquerading as a document) for properly understanding the cognitive content that lies behind a presentation [19,20]. A forest plot conforms to a universally understood graphical vocabulary whereby the 'result' and its statistical significance can instantly be read by anyone familiar with the convention. In contrast, there is no sense in which the harvest plot can be interpreted as showing a 'statistically significant' result; rather, it helps to illustrate the distribution of the evidence, such as it is, in terms of which of the competing hypotheses are more or less strongly supported.

One particular example of the need for an accompanying narrative synthesis is that the evidence collected under the central column – supporting the null hypothesis of no gradient in effectiveness – is likely to include several types of 'null' evidence: studies which have genuinely and robustly demonstrated the absence of a gradient; underpowered or poorly-executed studies which were highly unlikely to detect a gradient even if such were present; or studies with internally conflicting results which have been treated as cancelling each other out for the purpose of populating the matrix. We have not yet found a satisfactory way of disentangling this diversity of 'null' evidence other than in the accompanying narrative synthesis.

## Conclusion

The harvest plot is a novel and useful aid to synthesising evidence about the differential effects of complex, heterogeneous, population-level interventions. It combines the visual immediacy of the conventional forest plot with a much more inclusive, hypothesis-testing approach to summarising the distribution of the best available evidence across multiple simultaneous dimensions of inequality. The method is suitable for adaptation to a variety of questions in evidence synthesis. We therefore invite colleagues to consider applying and adapting the harvest plot as a component of the processes of synthesising and reporting the findings of systematic reviews of the differential effects of other complex interventions.

## Competing interests

The authors declare that they have no competing interests.

## Authors' contributions

DO, MP, AS and MW designed the overall study for which the methods, results, and authors' contributions have been reported elsewhere (Thomas S, Fayter D, Misso K, Ogilvie D, Petticrew M, Sowden A, Whitehead M, Worthy G. Population tobacco control interventions and their effects on social inequalities in smoking: systematic review, submitted). DO designed the matrices, populated them with data, and wrote the paper. DF coined the term 'harvest plot'. All authors contributed to the analysis and interpretation of the data and the critical revision of the manuscript and read and approved the final manuscript.

## Pre-publication history

The pre-publication history for this paper can be accessed here:



## Supplementary Material

Additional file 1 Study design and other methodological criteria. Details of the criteria used to weight and annotate the marks for each study. Click here for file

## References

[B1] Higgins J, Green S (2006). Analysing and presenting results. Cochrane handbook for systematic reviews of interventions 4.2.6 [updated September 2006]; section 8. The Cochrane Library.

[B2] Deeks J, Khan K, Song F, Popay J, Nixon J, Kleijnen J, Khan K, Kleijnen J, Glanville J, Sowden A, Kleijnen J (2001). Stage II: Conducting the review. Phase 7: Data synthesis. Undertaking systematic reviews of research on effectiveness: CRD's guidance for those carrying out or commissioning reviews.

[B3] Lewis S, Clarke M (2001). Forest plots: trying to see the wood and the trees. BMJ.

[B4] Slavin R (1995). Best evidence synthesis: an intelligent alternative to meta-analysis. J Clin Epidemiol.

[B5] Jackson N, Waters E, the Guidelines for Systematic Reviews of Health Promotion and Public Health Interventions Taskforce (eds.) (2005). Guidelines for systematic reviews of health promotion and public health interventions Version 12.

[B6] Popay J, Roberts H, Sowden A, Petticrew M, Arai L, Rodgers M, Britten N, Roen K, Duffy S (2006). Guidance on the conduct of narrative synthesis in systematic reviews: a product from the ESRC Methods Programme.

[B7] Lucas P, Baird J, Arai L, Law C, Roberts H (2007). Worked examples of alternative methods for the synthesis of qualitative and quantitative research in systematic reviews. BMC Med Res Methodol.

[B8] Tugwell P, Petticrew M, Robinson V, Kristjansson E, Maxwell L, the Cochrane Equity Field Editorial Team (2006). Cochrane and Campbell Collaborations, and health equity. Lancet.

[B9] Macintyre S, Chalmers I, Horton R, Smith R (2001). Using evidence to inform health policy: case study. BMJ.

[B10] Evans T, Brown H (2003). Road traffic crashes: operationalizing equity in the context of health sector reform. Inj Control Saf Promot.

[B11] Briss P, Zaza S, Pappaioanou M, Fielding J, Wright-De Agüero L, Truman B, Hopkins D, Dolan-Mullen P, Thompson R, Woolf S, Carande-Kulis V, Anderson L, Hinman A, McQueen D, Teutsch S, Harris J, Task Force on Community Preventive Services (2000). Developing an evidence-based guide to community preventive services – methods. Am J Prev Med.

[B12] Thomas H (2003). Quality assessment tool for quantitative studies.

[B13] Chaloupka F, Wechsler H (1995). Price, tobacco control policies and smoking among young adults.

[B14] Lewit E, Hyland A, Kerrebrock N, Cummings KM (1997). Price, public policy, and smoking in young people. Tob Control.

[B15] Miles M, Huberman A (1994). Qualitative data analysis: an expanded sourcebook.

[B16] Fienberg S (1979). Graphical methods in statistics. Am Stat.

[B17] Wainer H (1992). Understanding graphs and tables. Educ Res.

[B18] Jackson N (2005). Systematic reviews of health promotion and public health interventions.

[B19] "Slideuments" and the catch-22 for conference speakers. http://www.presentationzen.com/presentationzen/2006/04/slideuments_and.html.

[B20] Tufte E (2003). The cognitive style of PowerPoint: pitching out corrupts within.

